# Processive DNA Demethylation via DNA Deaminase-Induced Lesion Resolution

**DOI:** 10.1371/journal.pone.0097754

**Published:** 2014-07-15

**Authors:** Don-Marc Franchini, Chun-Fung Chan, Hugh Morgan, Elisabetta Incorvaia, Gopinath Rangam, Wendy Dean, Fatima Santos, Wolf Reik, Svend K. Petersen-Mahrt

**Affiliations:** 1 DNA Editing in Immunity and Epigenetics, IFOM-Fondazione Instituto FIRC di Oncologia Molecolare, Milano, Italy; 2 DNA Editing Lab, Clare Hall Laboratories, London Research Institute, South Mimms, United Kingdom; 3 Laboratory of Developmental Genetics and Imprinting, The Babraham Institute, Cambridge, United Kingdom; 4 Centre for Trophoblast Research, University of Cambridge, Cambridge, United Kingdom; Institut de Recherches Cliniques de Montréal (IRCM), Canada

## Abstract

Base modifications of cytosine are an important aspect of chromatin biology, as they can directly regulate gene expression, while DNA repair ensures that those modifications retain genome integrity. Here we characterize how cytosine DNA deaminase AID can initiate DNA demethylation. *In vitro*, AID initiated targeted DNA demethylation of methyl CpGs when in combination with DNA repair competent extracts. Mechanistically, this is achieved by inducing base alterations at or near methyl-cytosine, with the lesion being resolved either via single base substitution or a more efficient processive polymerase dependent repair. The biochemical findings are recapitulated in an *in vivo* transgenic targeting assay, and provide the genetic support of the molecular insight into DNA demethylation. This targeting approach supports the hypothesis that mCpG DNA demethylation can proceed via various pathways and mCpGs do not have to be targeted to be demethylated.

## Introduction

Cytosine methylation and its oxidative variants are important mammalian DNA modifications, playing key roles for the maintenance of genomic stability and cellular identity by controlling gene expression, genomic imprinting, X chromosome inactivation, and silencing of transposable elements [1], [2]. Concurrently, DNA demethylation is required during early development, in somatic cells during differentiation, and for cellular reprogramming, stressing the reversible nature of DNA methylation [2], [3]. Yet, while the process of establishing and maintenance of DNA methylation by DNA methyltransferases (DNMTs) is well characterized, understanding the molecular mechanism underlying active DNA demethylation is only in its beginnings [3]. In mammals, proposed mechanisms for DNA demethylation involve modification of the methylcytosine (5mC), followed by DNA repair dependent cytosine (dC) substitution [1], [2], [3], [4], [5], [6]. 5mC-modifying enzymes include hydroxylases and deaminases. The Ten-eleven translocation (TET) family proteins hydroxylate 5mC [7], [8], with the resulting hydroxymethylcytosine (5hmC) either oxidized [8], [9] and/or replaced with cytosine - possibly via base excision repair (BER) [10]. Proteins of the activation-induced deaminase (AID)/apolipoprotein B mRNA-editing enzyme complex (APOBEC) family can deaminate 5mC to thymine (dT) [6], creating a dT:dG mismatch, which can be repaired back to a cytosine via the BER pathway [11], [12]. Although there is a debate on the extent of AID's involvement in DNA demethylation, a number of publications have identified important genetic links. These include DNA methylation alterations in zebrafish after addition and removal of AID [12], loss of global DNA demethylation in AID deficient mice [13], lack of complete reprogramming from AID deficient cells during heterokaryon fusions [14], and inefficient iPS formation from AID -/- cells [15]. On the other hand, from the recent literature it is clear that other enzymes or pathways (e.g. GADD45 or Nucleotide Excision Repair - NER) are also involved in DNA demethylation [4], [5], hence it is unlikely for AID (or family members) to be responsible for all observed DNA demethylation events.

AID was initially described as being essential for the diversification of immunoglobulin (Ig) genes. In activated B cells AID deaminates, on single stranded DNA (ssDNA), cytosine residues to uracil (dU) [16]. Depending on multiple factors, such as the cell state, chromatin state, and location, the dU lesions will lead to repair, or point mutations and DNA recombination. In the Ig locus, dUs are necessary to induce antibody affinity maturation via somatic hypermutation (SHM), and change of antibody effector functions via class switch recombination (CSR) [17], [18]. The exact molecular mechanisms are beginning to be revealed, with proteins from DNA repair pathways [including BER and mismatch repair (MMR)] playing a necessary role during SHM [19], [20], [21].

Here we begin to delineate the molecular mechanisms of AID-induced lesion processing - *in vivo* and *in vitro* - leading to repair and DNA demethylation. We utilized our recently developed GAL4 targeting in an *in vitro Xenopus* egg extract system [22] as well as a transgenic mouse approach. DNA demethylation was analyzed either from controlled methylated plasmids (*in vitro*) or the differentially methylated region (DMR) of the imprinted H19 gene (*in vivo*). Both approaches demonstrated that AID-induced DNA demethylation can be mediated by BER (UNG-dependent and independent) and DNA repair pathways requiring processive DNA polymerases. Furthermore, the use of processive DNA polymerase dependent pathways does not necessitate targeting 5mCpGs for demethylation.

## Materials and Methods

### Generation of transgenic mice

All experimental procedures were conducted under licenses by the Home Office (UK) in accordance with the Animals (Scientific Procedures) Act 1986. The AID coding region up to 510 bp (excluding the nuclear export sequence at 550 to 596 bp at the C-terminus) was amplified from oocyte cDNA using primers *BamH*I-AIDF (GGATCCATGGACAGCCTTCTGATGAAGCAA) and *EcoR*I-AIDR (GAATTCCAGAATTTTCATGTAGCCCTTCCCAG) to generate *BamH*I-AID-*EcoR*I PCR product. This was inserted into a vector containing the CMV promoter driving the GAL4 DNA binding domain [23] (a kind gift from François Fuks, Free University of Brussels, Belgium) to generate the CMV GAL4-AID plasmid. Plasmids CMV GAL4-ΔAID1 and CMV GAL4-ΔAID2 were derived from CMV GAL4-AID by in vitro mutagenesis. Inserts were excised from the plasmids by *Nru*I and *Dra*III restriction enzyme digestion, were purified (Qiagen), and used for microinjection into F1 (C57Bl/6J x CBA/CA) x F1 fertilised zygotes, which were subsequently transferred into pseudopregnant mothers. Genotyping of transgenic mice was carried out by PCR on DNAs from tail biopsies, using primers GAL4AID (s) GTCCAGTGAGCAGGAGGTG and GAL4AID (as) CCAAAGAAAAACCGAAGTGC which are in the GAL4 and AID regions respectively such that GAL4-AID transgenes are specifically detected without amplifying the endogenous *aid* gene.

### RNA expression of transgenes

Total RNA was extracted from different embryonic and postnatal tissues with the RNeasy mini/midi kit (Qiagen). cDNA was synthesised by using SuperScript II reverse transcriptase (Invitrogen). The efficiency of cDNA synthesis was evaluated by PCR for Hprt. To ensure there is no DNA contamination, reactions without reverse transcriptase were always done in parallel. Expression of GAL4-AID transcripts was analyzed by RT-PCR using primers in the GAL4 region (s: AAGTGCGCCAAGTGTCTGAA) and AID region (as: CAGCCAGACTTGTTGCGAAG) to prevent amplification of endogenous AID transcripts. Quantitative real time PCR experiments were performed in triplicate with an ABI PRISM 7700 Thermocycler (Applied Biosystems); the relative quantification, amplification efficiencies, and comparative method of relative quantification were done according to instructions supplied by Qiagen.

### GAL4 staining of zygotes

Fertilized oocytes were washed in PBS, and after fixation in 4% paraformaldehyde in PBS for 15 minutes, the zonae pellucidae were removed with Tyrode's Solution Acidic (Sigma) and the oocytes permeabilised with 0.2% Triton X-100 in PBS for 1 h, at room temperature. After blocking in 0.05% Tween-20 in PBS containing 1% BSA (B-PBS) overnight at 4°C, the oocytes were incubated with anti-GAL4 rabbit polyclonal antibody (Santa Cruz, sc-577) diluted 1:30 (B-PBS) for 3 hours at room temperature. Detection was achieved using goat α-rabbit IgG-Alexa (Molecular Probes) as secondary antibody. DNA was stained with DAPI (5 µg/ml) and all samples were mounted in Slow Fade (Molecular Probes). Image acquisition was performed with a LSM 510 Meta confocal laser scanning microscope (Carl Zeiss) equipped with a “Plan-Apochromat” 63x/1.40 DIC oil-immersion objective. Final pictures were obtained by Z-stack projection of serial sections (800×800, pixel size; z-step, 0.46 µm).

### DNA Methylation Assays

Bisulfite sequencing of genomic DNA. gDNA from tissues were digested with *Nco*I restriction enzyme, alkaline denatured, and treated with bisulfite as described [24]. All three regions (Bi-2, -3, -4) were amplified by the use of a nested PCR strategy. Sequences of primers are in Table S1. The PCR products were cloned (TA Cloning Kit, Invitrogen) and sequenced using the Applied Biosystems sequencing system.

CpG methylation status of the plasmid after the in vitro assay was monitored by bisulfite sequencing with the EZ DNA Methylation-GoldTM kit (Zymo Research). Bisulfite-treated DNA was amplified with the Zymo TaqTM PreMix (Zymo Research) and the pair of primers 6782 5′-GTTTTGATTGGGATAAAATTATTGT-3′/6781 5′-CTCACCTACCTCCTTACTAAACGAC-3′ amplifying sequence BS. PCR products were separated on 1% agarose gels, purified by Qiaquick Gel Purification (Qiagen), cloned into carrier plasmids by using the TOPO TA Cloning Kit (Invitrogen) and sequenced.

### Expression Vectors and protein expression

Human His-Tagged GAL4-AID coding vector was constructed by inserting the DNA-binding domain of GAL4 into the *Nco*I restriction site of the pET30 derived vector encoding for AID with a C-terminal His tag (described in Morgan *et al*. [6]). Site-directed mutagenesis was used to create catalytic inactive AID C89R. Wt and mutant GAL4-AID proteins were prepared as described for untagged AID [25]. Mouse GAL4-AIDΔC was cloned the same way and its mutation activity tested in a standard rifampicin mutation assay [25].

### *In vitro* resolution assay (IVR)

The details of this assay have been published [22]. Briefly: o. 5 µg of GAL4-AID (wt or mutant) were incubated with 0.1 pmol of the plasmid at 37°C for 30 min in buffer IVR. The target plasmid was either unmethylated or *in vitro* methylated with the *M.Sss*I methyltransferase (New England Biolabs). The repair reaction was performed by adding 150 µg of *Xenopus laevis* egg extract (FE) [26] supplemented with 5 µg aphidicolin (Sigma-Aldrich), 0.05 mM dNTP (without dCTP or dATP), and 0.05 mM biotinylated-dCTP or biotinylated-dATP, and incubated at 23°C for 30 min. When specified, 0.75 U UNG inhibitor (UGI - New England Biolabs) was added to the FE prior to plasmid addition. Treated DNA was purified via Qiagen Mini-prep (saved as input), isolated on streptavidin magnetic beads (Invitrogen-Dynal), collected in 100 µl TE, and 2 µl of this bead mixture subjected to real-time PCR. PCR reactions (20 µl) contained 10 µl of the LightCycler 480 SYBR Green I master mix (Roche Applied Sciences) and primers (1512 GGCCTAACTGGCCGGTAC Rev - 1518 GTCCACCTCGATATGTGC). The reaction was monitored in a LightCycler 480 Real-Time PCR System (Roche Applied Sciences); with the ‘input’ DNA analyzed in parallel as reference. C_t_ values for the biotinylated-DNA were correlated to the C_t_ values for the input DNA. Results were presented either as relative (fold-change) or absolute (% of input) quantification [22]. For fold-change, all samples were correlated to their input and then the FE alone sample (or another specified sample) was used as reference and set to one. Alternatively, in the % of input analysis the C_t_ qPCR values of input and output were converted to an absolute amount of DNA based on a standard curve, with the amount of isolated biotinylated-plasmid being expressed as a percentage of the initial amount of plasmid (input).

### Statistical analysis

We performed a paired two tailed Student T-test on the values for the bisulfite analysis and *Dpn*I analysis of AID-induced m6A-demethylation. Significance was indicated for p<0.05. We also performed chi-squared analysis of the bisulfite data. Here, the untreated (G-AID + FE) demethylation were set as the expected and the treated (G-AID + FE plus Ugi) as the observed. Chi-squared values were obtained using the Yates correction for using only one degree of freedom. Significance was indicated for p<0.01.

## Results

### *In vitro* AID-induced lesion repair

Although it is not fully clear to what extent AID is involved in DNA demethylation, we want to delineate the molecular mechanism of DNA demethylation from DNA deaminase-induced lesions. For this we are utilizing both an *in vitro* as well as an *in vivo* approach. The *in vitro* assay allowed us to obtain qualitative and quantitative readouts, while at the same time providing a means to control all aspects of the AID-induced DNA demethylation. While the transgenic mouse system recapitulated the *in vitro* results and provided important genetic insight into AID-induced demethylation *in vivo*.

To develop an *in vitro* DNA demethylation system, we modified our *in vitro* resolution (IVR) assay (Figure S1 and [22]). Briefly, a bacterially produced GAL4-AID (G-AID) fusion protein was targeted to GAL4 DNA-binding sites (UAS) on a methylated (*Sss*I) supercoiled plasmid. Once bound, AID deaminated dCs to dUs in the context of ssDNA. Subsequently, the reaction was added to *Xenopus laevis* egg extracts (FE). Here, DNA topoisomerase I and II relaxed the plasmid forming dU:dG lesions. This provided a substrate for various DNA repair pathways, including short-patch (SP-) BER and long-patch repair (MMR, long-patch (LP-) BER). DNA repair was monitored via incorporation of biotinylated-dCTP (bio-dC) or biotinylated-dATP (bio-dA). After streptavidin-bead isolation biotinylated plasmids recovery was quantitated with qPCR (quantitative real-time PCR). qPCR values from control reactions as well as the ratio of input to recovered plasmid was used to quantify AID and FE activity.

The results can be represented as bar-graphs and demonstrate that catalytic active G-AID and a functional FE are required for full activity [22]. The full details and consequences of the IVR, which are also relevant for the current work, can be found in our previous publication [22], briefly: a) the distance between GAL4 binding and AID-induced lesion was not significantly relevant; b) the presence of biotin did not alter the readout as they are represented as relative to each other; c) the activity of GAL4-AID is not limited to just a few cytosines near the GAL4 binding site, but dispersed throughout the plasmid, and hence biotin incorporation, streptavidin isolation, and qPCR results are based on a population analysis with minimal local effects off-setting each other; d) the avoidance of enzyme excess over substrate (a drawback on a number of previous *in vitro* assays using AID [27]) precluded those circumstances where AID can induce multiple deaminations in quick succession; and e) in the absence of FE or presence of only the GAL4 binding domain (GBD) biotin was not incorporated [22].

Furthermore, removing the GAL4 DNA binding domain from AID had reduced biotin incorporation, which was alleviated once the incubation time of AID (no GAL4) and plasmid was extended [22]. This indicated that the GAL4 domain simply enhanced the molecular crowding aspect of the reaction by allowing AID to finds its DNA target more efficiently. Since our assay was designed to understand the mechanisms downstream of AID-induced lesions, the GAL4 DNA binding domain only served as a means to enhance the speed and accuracy of the reaction but not the targeting. The *in vivo* mechanism of AID-targeting had been addressed previously by us [28], and we did not want to add this extra complexity to a biochemical and mechanistic analysis of post-AID lesion events.

From our work on unmethylated substrates for AID-induced damage resolution [22], we were able to demonstrate that the lesions stimulate various DNA repair pathways. They included BER (SP-BER and LP-BER) and MMR (non-canonical), which directly recapitulated usage of those DNA repair pathways that arise from AID-induced lesions during immunoglobulin diversification *in vivo* [19].

### IVR development for AID-induced demethylation

**Figure 1:**
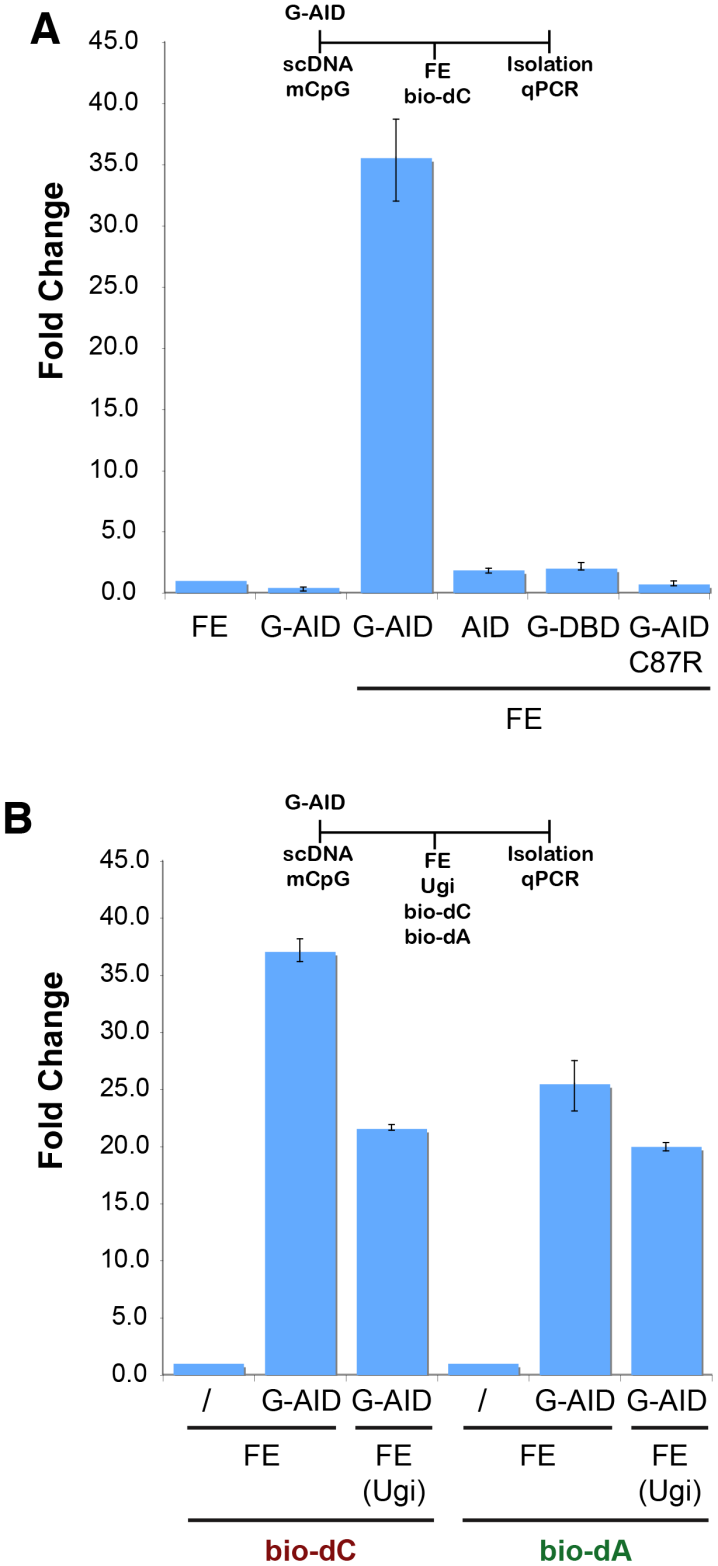
AID-induced lesions on a methylated substrate are resolved by DNA repair. (**A**) AID-induced lesions result in bio-dC incorporation. G-AID was incubated with a pre-methylated substrate (*M.Sss*I - Figure S2) for 30 min and then added to FE. Repaired plasmids (bio-dC labelled) were isolated and quantified by qPCR. The bars represent the ratio (Fold change) of the amount of recovered plasmids from reactions carried out in the presence of G-AID, untagged AID (AID), the GAL4 DNA binding domain (G-DBD), or the mutant G-AID C87R versus levels of plasmids recovered from reactions that did not contain G-AID (FE, set to 1). Error bars indicate ± standard deviation (SD, n = 3). Line schematics for all IVR assays are shown above the graphs, and they indicate the order of addition of substrates/proteins/nucleotides/extract/etc. or treatments. (**B**) AID-induced lesion resolution on methylated targets involves BER and processive DNA polymerases-dependent repair. IVR reactions were performed as in (A), containing either bio-dC or bio-dA during the resolution phase of the IVR. Where indicated, FE was treated with Ugi prior to addition of the plasmids (FE (Ugi)). The bars depict the levels of plasmids (bio-dC or bio-dA labelled) that were recovered from the individual reactions. Samples were normalized to FE reactions without G-AID (FE) and were set to 1. Error bars indicate ± standard deviation (SD, n = 3).

A current model for AID-induced DNA demethylation suggests that the T:G mismatch, created by AID upon deamination of 5mC, is processed via the BER pathway [6], [11], [12]. To better understand which DNA repair pathway is involved, we modified our IVR assay using a methylated substrate (Figure S1), where the plasmid DNA was fully methylated at 5mCpG with M.*Sss*I (Figure S2). Because M.*Sss*I treatment can introduce DNA damage itself, stemming from lack of the co-factor SAM or buffer composition, utilization of various mock treatments (Figure S2A) demonstrated that the observed IVR result (Figure S1) was only dependent on the AID activity acting on the methylated substrates. The M.*SssI* treatment did not change plasmid topology significantly (Figure S2B), nor did it alter the efficacy of the AID-IVR assay (Figure S2C). As seen in Figure 1A, just as for the unmethylated substrate [22], the methylated plasmid was acted upon by catalytic G-AID and repaired in the IVR. Neither the catalytic dead AID (G-AIDm C87R - [29]), the GAL4 DNA binding domain (G-DBD), nor the untargeted AID (AID) were able to induce DNA repair on the methylated plasmid.

### BER and other DNA repair pathways act during DNA demethylation

In our related work on unmethylated substrates [22], we were able to demonstrate that the AID-induced DNA damage can be resolved via various DNA repair pathways using the IVR system. Applying the same approach here, we demonstrate that with a methylated plasmid different types of DNA repair pathways are acting on the AID-induced lesions. When the BER inhibitor Ugi was added to the IVR, we observed a reduction without complete inhibition (Figure 1B). Although the use of Ugi could have minor side effects, the peptide has been extensively studied and characterized and shown remarkable specificity for inhibiting UNG2 (the predominant BER protein acting on dUs) [30], [31] without affecting other uracil DNA glycosylases (e.g. TDG, MBD4, SMUG). This indicated that although UNG-dependent BER is important in AID-induced lesions resolution of methylated plasmids, other DNA repair pathways are also playing a role. During SP-BER the dU:dG (or dT:dG) lesion is repaired with the incorporation of a single dC, resulting in a dC:dG base pair. Processive polymerase-dependent repair pathways (e.g. LP-BER, MMR), not only resynthesize the lesion, but also incorporate nucleotides that are downstream of the initial dU. These pathways can be detected in the IVR by addition of biotinylated-dA (Figure 1B), where a significant amount of biotin incorporation can be seen after treatment with G-AID and FE. Importantly, by adding Ugi, we are able to discern between UNG-dependent LP-BER and other processive polymerase-dependent DNA repair pathways, such as non-classical MMR [21].

### AID-induced single and processive DNA demethylation

**Figure 2:**
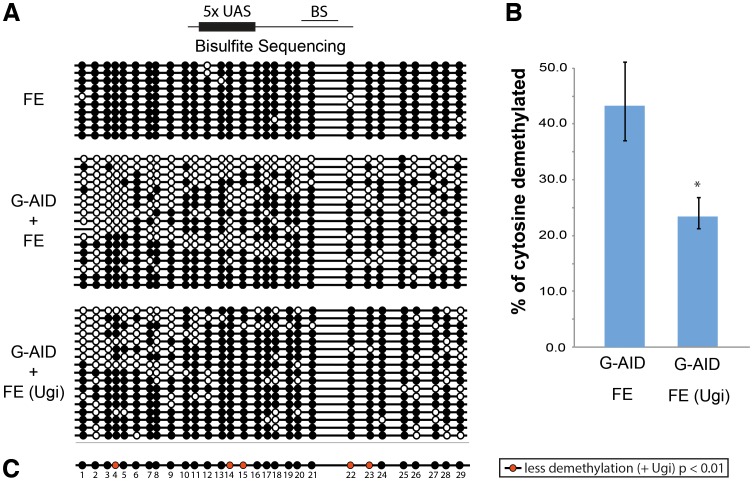
AID-induced lesion repair results in demethylation. (**A**) AID induces demethylation of 5mCpG *in vitro*. Methylated plasmids from IVR reactions in Figure 1 were subjected to bisulfite sequence analysis of regions BS, 3' of the GAL4 binding site. Methylation was monitored from IVR-samples performed with GAL4-AID (G-AID + FE) or without (FE), and in the presence of Ugi (G-AID + FE (Ugi)). White and black circles represent unmethylated and methylated cytosines, respectively. (**B**) Quantification of 5mC demethylated after the IVR assay from (A). Error bars indicate ± SD (n = 3); a t-test showed a significant difference between the two groups (p<0.05). (**C**) Graphical representation of those CpGs that were significantly inhibited by Ugi treatment for resolving AID-induced demethylation in the IVR. Each CpG (1 - 29) was analyzed using chi-squared analysis, and red circles indicate p<0.01.

Aside from identifying the various DNA repair pathways acting on the AID-induced lesions of a methylated substrate, we also determined the extent of local DNA demethylation. Using bisulfite analysis of a region downstream of the GAL4 DNA-binding site, we identified both single site demethylation as well as consecutive (processive) demethylation events (Figure 2A), with AID activity leading to 43% cytosine demethylation (Figure 2B). One should note that although the DNA is CpG methylated, unmethylated dCs outside a 5mCpG context are still substrates for AID-induced deamination. When we treated the FE with Ugi, to inhibit UNG2 dependent BER, bisulfite analysis showed a significant decrease in the efficiency of the extract to induce DNA demethylation (Figure 2A & B). This strongly suggested that AID can induce DNA demethylation by acting on dCs, since dUs are the only substrate for UNG2. Furthermore, if dCs are deaminated to dUs and UNG2 lesion processing leads to 5mCpG demethylation, then LP-BER plays a role in local DNA demethylation.

### Local 5mCpG context prescribes DNA repair pathway choice for DNA demethylation

Given the multitudes of DNA repair pathways acting on AID-induced lesions, we wanted to determine if there is a preference of DNA repair pathway choice for demethylation of individual 5mCpGs. To this end, we statistically analyzed the difference of demethylation frequency of each CpG in the G-AID + FE and G-AID + FE (Ugi) samples. Using chi-squared analysis we were able to identify 3 regions (CpG 4, 14–15, and 23–24) that where, upon treatment of the FE with Ugi, significantly precluded to undergo DNA demethylation. Furthermore, some regions (CpG 7, 24, 26) had less significant inhibition of DNA demethylation, while others (CpG 1–2, 8–9, 11, 18, and 29) were refractory for the Ugi inhibition.

Overall, these results indicate that AID-induced DNA demethylation can be mediated via different DNA repair pathways, which are either UNG-dependent (5mC independent), UNG-independent (e.g. TDG), and/or are repaired beyond the initial lesion via a processive DNA polymerase.

### Induced demethylation does not require CpG recognition

We have previously determined that AID deaminates unmethylated cytosines about 5–10 times more efficiently than methylated ones [6]. Furthermore, the IVR target plasmid contains 844 dCs in the context of WRC (A/T,A/G,C - the preferred AID target), yet only 161 are in the context of WRCpG. Hence given the random targeting of G-AID, it is more likely that both dU:dG and dT:dG mismatches are present on an AID treated methylated-substrate, rather than just dT:dG. This mixture also precluded the identification of a strict requirement for only 5mC deamination leading to DNA demethylation. Also, the experiments with bio-dA or/and Ugi inhibition (Figure 1B) suggested that DNA repair processed and incorporated bio-dC/bio-dA beyond the initial AID-induced lesion. All of this suggests that if a 5mC was in proximity to a deaminated dC (dU) and processive DNA polymerase repair replaced the 5mC with dC, then DNA demethylation proceeded without having initially targeted the 5mCpG.

**Figure 3:**
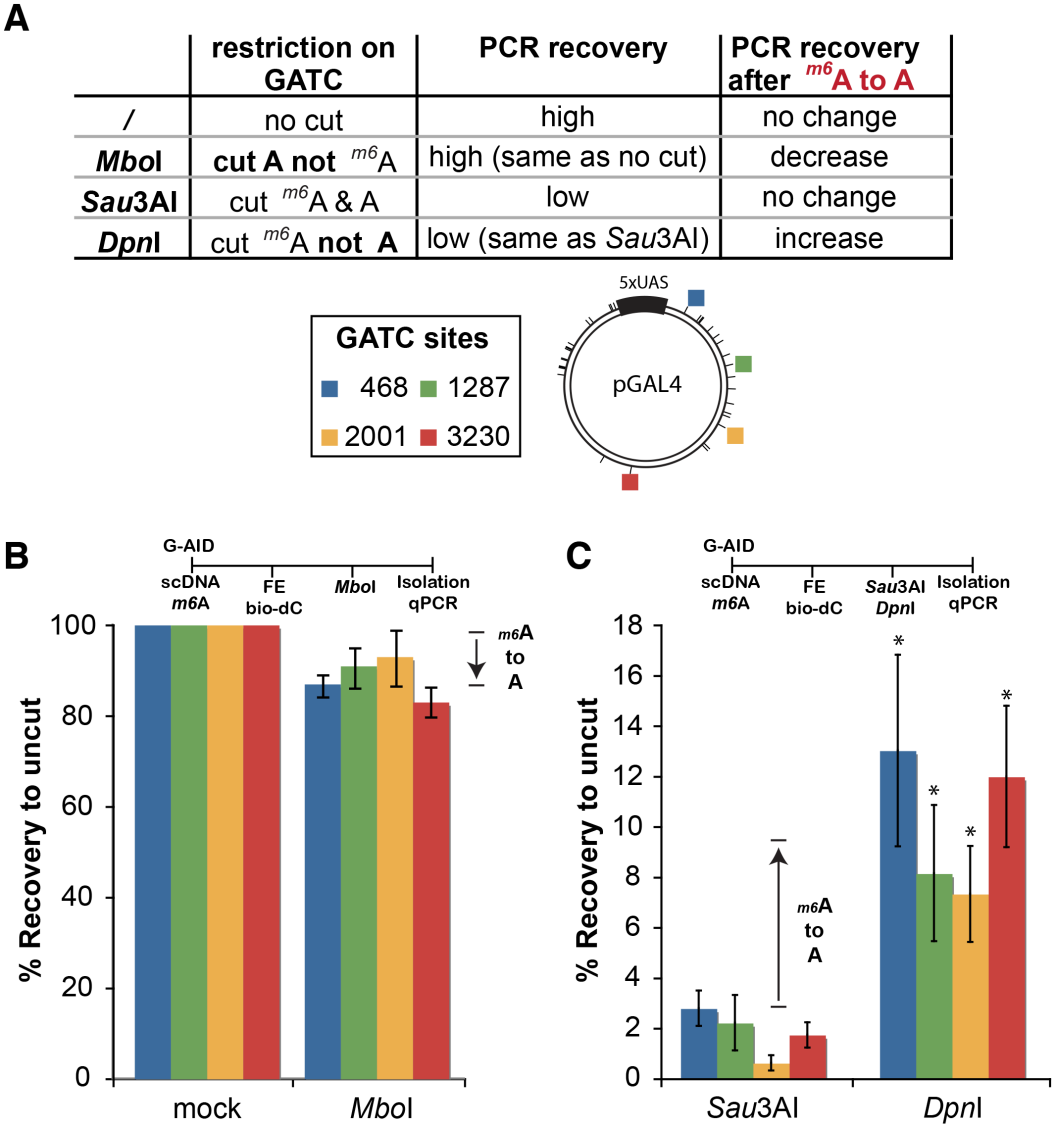
AID induces demethylation independent of the nucleotide context. (**A**) The table summarizes whether methylation of the adenosine (*m6*A) in GATC sites influences cutting by the restriction enzymes *Mbo*I, *Sau3A*I or *Dpn*I. On the left is the preference of cutting depending on the state of adenosine-methylation. In the middle the absolute PCR recovery after digestion is indicated. The outcome of the PCR recovery of digested DNA after AID-induced demethylating *m6*A is indicated on the right. (**B**) (**C**) AID induces demethylation of methyl-adenosine. Supercoiled plasmids (*m6A(+*) as isolated from standard *E. coli*) were subjected to IVR assays containing GAL4-AID as in Figure 1. Streptavidin-isolated plasmids were either undigested (mock) or incubated with the restriction enzymes *Mbo*I, *Dpn*I, and *Sau3A*I (NEB, USA) prior to qPCR amplification. The different GATC sites (positions: 468, 1287, 2001 and 3230) are indicated by colors and marked on the plasmid map; there are 37 GATC sites within the plasmid substrate. IVR results were quantitated by setting the uncut G-AID treated samples to 100%. Correction factors based on FE alone activity (for *Mbo*I) or Fe + *Sau3A*I activity (for *Dpn*I) was determined from Figure S3 and applied to the G-AID induced recoveries. Loss of *m6*A is shown in (**B**) by a decrease in recovery after *Mbo*I cutting versus uncut, and by an increase in recovery after *Dpn*I digest (**C**). A standard t-test indicated that after G-AID treatment all sites showed a significant difference between *Sau3A*I and *Dpn*I cutting; (p<0.05 - indicated as *), (n≥3).

To prove this non-targeting aspect of DNA demethylation we demonstrated that G-AID can induce methyl-adenosine demethylation on the plasmid via targeting dCs. Plasmid DNA isolated from common molecular biology grade *E. coli* hosts is methylated on adenosine in the context of G*m6*ATC (dam methylation - *m6*A). In the IVR, dam methylation did not alter G-AID-induced activity on cytosines (comparing plasmids from *dam* (−) or (+) hosts - data not shown), indicating that the FE did not recognize *m6*A DNA base as a lesion. We analyzed 4 different G*m6*ATC sites in the plasmid (position 468, 1287, 2001, and 3230) for the effect of AID-induced *m6*A demethylation (Figure 3A), with the distribution of each site on the plasmid not biasing the IVR results [22]. After G-AID and FE treatment and prior to qPCR, the plasmids were restriction-digested by either of three isoschizomers recognizing GATC: *Mbo*I (sensitive to *m6*A), *Sau3A*I (resistant to *m6*A), or *Dpn*I (requiring *m6*A); cutting the GATC site prevents PCR amplification and reduces the signal. Therefore, *m6*A containing plasmids are digested (less recovery) by *Sau3*AI and *Dpn*I, while they are resistant (more recovery) to *Mbo*I cleavage. A decrease in resistance after *Mbo*I cutting and enhanced resistance after *Dpn*I cutting is a reflection of the incorporation of dA during AID-induced repair, and therefore demonstrates *m6*A demethylation.

We previously described that FE alone can induce minute amounts of DNA repair [22], and hence induce 6mA demethylation on its own. As seen in Figure S3, FE alone (no G-AID) treated substrates showed some difference between *Mbo*I and uncut, or *Dpn*I and *Sau3A*I digestion. The ratios of the differences were then used as a correction factor to determine AID-induced m6A demethylation. In Figure 3B and 3C, uncut G-AID treated samples were set to 100% and G-AID induced demethylation represented as % recovery to uncut. *Mbo*I reduced the efficacy of PCR amplification when compared to the uncut sample, indicating that the methylation of the four analyzed *m6A* sites had been lost. This AID-dependent demethylation of *m6A* sites was also observed when we restricted the plasmid with *Dpn*I and *Sau3A*I. *Dpn*I restriction was not as complete as *Sau3A*I (enhanced recovery), which also indicated a loss of *m6*A sites (Figure 3C).

These results clearly demonstrate that AID-induced lesions (dU) can be repaired with a processive polymerase-dependent repair system, leading to substitution of methylated adenosine by unmethylated adenosine. Therefore, DNA repair from AID-induced lesions is sufficient to induce demethylation without directly targeting the methylated base. TET modified 5hmC is not a target for direct AID induced deamination, as its bulky side-chain on C5 does not fit the active site of AID [32], nevertheless 5hmC can be removed from DNA by an AID-dependent mechanism, if AID-induced uracils are present near 5hmC prior to processive polymerase dependent DNA repair.

### *In vivo* targeting of AID induces local demethylation

**Figure 4:**
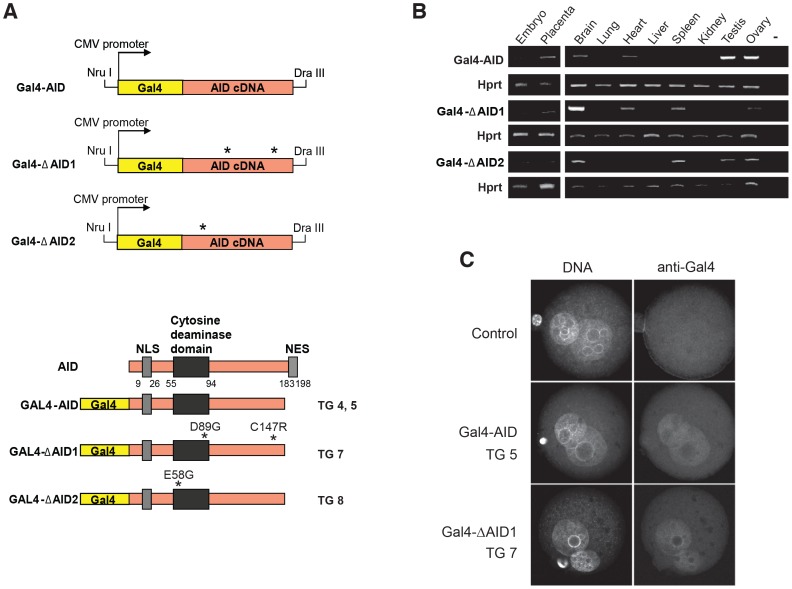
Structure of GAL4-AID transgenes and their expression. (**A**) GAL4-AID fusion cDNAs were inserted into a CMV promoter containing vector resulting in three transgene constructs (GAL4-AID, GAL4-ΔAID1, and GAL4-ΔAID2), which were excised from the plasmid backbone with *Nru*I and *Dra*III and microinjected into zygotes, resulting in transgenic strains TG 4 and 5, TG 7, and TG 8. Lower panel: The GAL4 DNA binding domain was fused to the AID cDNA lacking the C terminal nuclear export signal (NES). In addition to wild-type AID two mutant forms of AID cDNA, harbouring amino acid exchanges D89G and C147R, and E58G, respectively, were fused. Numbers refer to amino acids position in AID. (**B**) GAL4-AID expression in transgenic strains TG 5 (GAL4-AID), TG 7 (GAL4-ΔAID1), and TG 8 (CMV GAL4-ΔAID2) was monitored by RT-PCR. RNA samples from embryo, placenta and various adult tissues were analyzed. *Hprt* mRNA served as loading control. (**C**) Transgenic AID localizes in pronuclei of zygotes in TG 5 (Gal-4-AID) and TG 7 (GAL4-ΔAID1). GAL4-AID expression in zygotes was analyzed by immunofluorescence using an anti-GAL4 antibody. DNA was stained with DAPI. TG 5 and TG 7 resulted from crossing of transgenic mother with H19 DMR-UAS father, as a control crosses between C57Bl6 mother and H19 DMR-UAS father was used.

**Figure 5:**
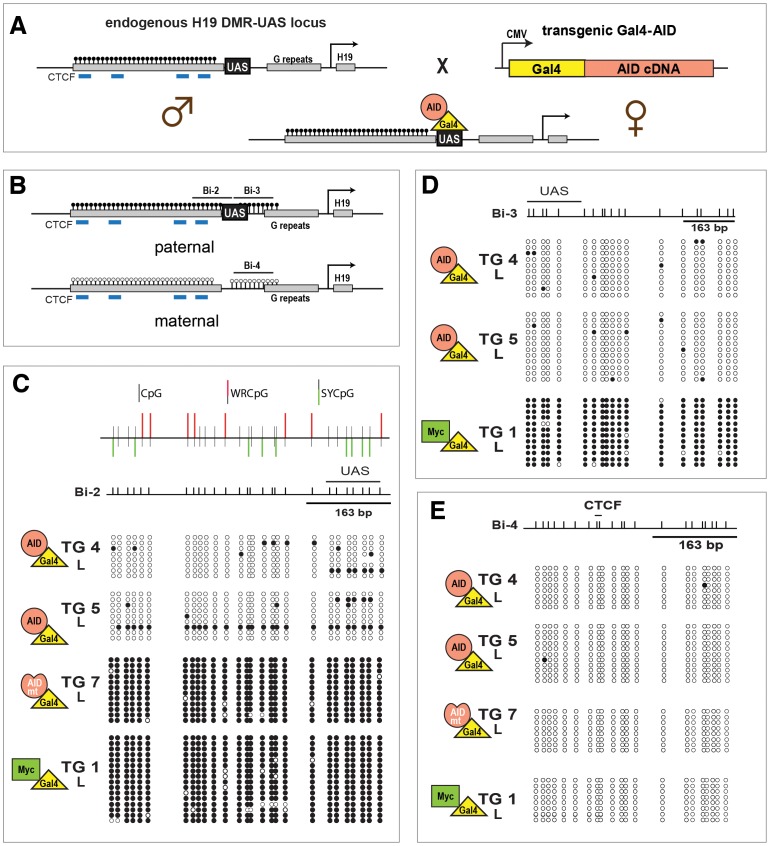
Targeting of GAL4-AID to the H19 DMR-UAS leads to demethylation. (**A**) Schematic of the paternal knock-in H19 DMR-UAS mouse and the maternal transgene GAL4-AID (where GAL4 is in yellow and AID in salmon) mouse. GAL4-AID transgenic females were crossed with H19 DMR-UAS homozygous male. (**B**) Methylation of the loci surrounding the UAS was analyzed by bisulfite sequencing. The regions analyzed by bisulfite sequencing (Bi-2, Bi-3, Bi-4), the CTCF binding sites (blue lines), the G repeats sequence, the transcription start site (black arrow) and the position of the UAS within the H19 DMR are depicted. (**C** –**E**) AID-induced methylation alterations at the Bi-2 (**C**) and Bi-3 (**D**), but not at Bi-4 (**E**) regions. DNA methylation of H19 DMR alleles was analyzed by bisulfite sequencing of DNA from neonatal liver (L) of transgenic offspring of GAL4-AID wt (TG 4 and TG 5), mutant GAL4-ΔAID (TG 7), or GAL4-myc (TG 1) crossed with H19 DMR-UAS. The top of the figure (**C**) represents the schematic of the context of the CpGs within the Bi-2 sequence: CpG with an AID hotspot motif WRC (in red, WRCpG) or in the cold spot motif SYC (in green, SYCpG). Filled circles represent methylated CpGs, open circles unmethylated ones. GAL4 is represented by a yellow triangle, AID wt by a salmon circle, AID mutant by a misformed salmon circle, and Myc by a green rectangle. Summary of the results are shown in Figure S5.

The *in vitro* data suggest that lesions induced by targeting of AID to a specific locus can induce DNA demethylation. In order to determine whether the *in vitro* observations also hold true *in vivo*, we used the GAL4-AID fusion and targeting strategy in a transgenic mouse approach. Here, a female transgenic GAL4-AID fusion protein mouse (Figure 4) was bred to a male mouse having the GAL4 binding sites (UAS) introduced into the first (of four) methylated H19-DMR (H19 DMR-UAS), using a previously developed transgenic strategy [33] (Figure 5A). This DMR (differential methylated region) was chosen because the paternal allele remains stably methylated in all embryonic and somatic tissues of the offspring. For the various GAL4-AID fusions (Figure 4A), transgenes were driven by the CMV promoter and the C-terminal region of AID, including the nuclear export signal, was deleted. Although deletion of the C-terminal region can alter AID turnover [34], [35], overall it can enhance nuclear localization without diminishing deaminase activity [36], [37]. We also generated two mutants of the same transgene: CMV GAL4-ΔAID1 carries two amino acid changes (D89G & C147R) and CMV GAL4-ΔAID2 has a single amino acid change (E58G - [29]) in the catalytic domain (Figure 4A). Both of the mutant AID proteins are severely reduced in their catalytic activity (Figure S4). In all mouse lines the transgene was expressed in various embryonic and postnatal tissues, as tested by RT-PCR (Figure 4B) and by immunofluorescence in zygotes (Figure 4C).

**Figure 6:**
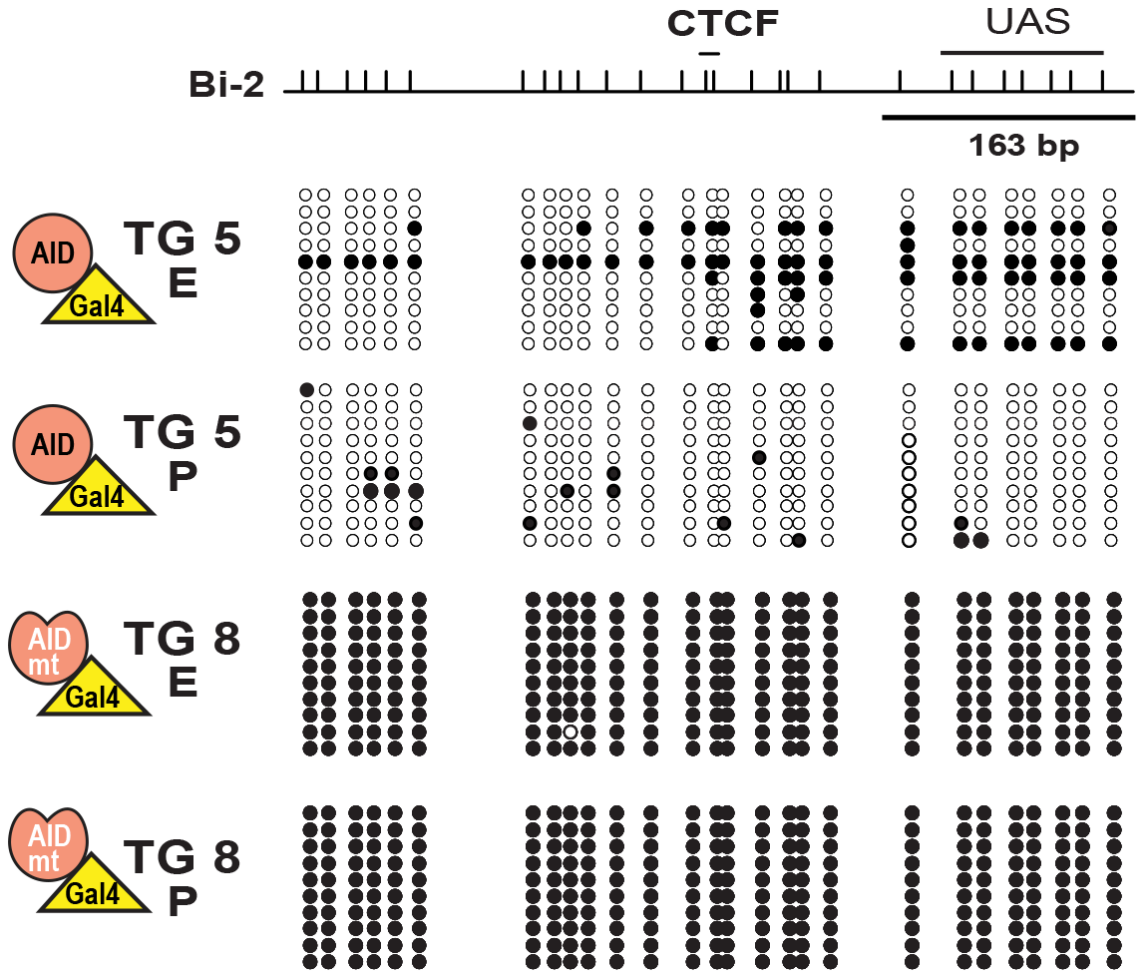
AID-induced demethylation in Embryo and Placenta. GAL4-AID wt (TG 5) or mutant (TG 8) transgenic females were crossed with H19 DMR-UAS homozygous males, and methylation was analyzed in embryos (E) and placentas (P) at E12.5 of transgene positive offspring. Bi-2 region was amplified and analyzed as in Figure 5. Filled circles represent methylated CpGs, open circles unmethylated ones.

We bred males harboring the H19 DMR-UAS locus with females carrying the GAL4-AID, GAL4-ΔAID mutants, or the previously described CMV GAL4-Myc [33] expressing transgenes, and determined the extent of DNA methylation in F1 offspring. Due to technical limitation of obtaining enough material from fertilized oocytes we could not perform bisulfite analysis on homogenous tissues right after fertilization (zygote). Hence, we choose to analyze tissue samples for methylation analysis of various regions surrounding the UAS (Bi-2, -3, -4) from neonatal liver (Figure 5). Since adult liver did not express the transgene itself (Figure 4B), it was likely that any observed demethylation had to occur in earlier stages of development. The H19 locus is an imprinted locus, with the paternal allele being methylated and the maternal allele unmethylated. Due to the genetic manipulations of the system regions Bi-2 & 3 can be amplified from the paternal allele, while region Bi-4 can only be amplified from the maternal allele (Figure 5B). As shown in Figure 5C, Bi-2 was significantly more demethylated in GAL4-AID than in GAL4-Myc mice. More importantly, the demethylation required AID catalytic activity, as transgenic mouse 7 (TG 7 - harboring a catalytic inactive GAL4-AID) did not show extensive DNA demethylation in this region. Bisulfite analysis of the Bi-3 region confirmed the results for the demethylation capacity of a catalytic active AID (Figure 5D and summarized in Figure S5A), where TG 4 and TG 5 induced over 95% demethylation. Loss of the methylation on the paternal allele may induce methylation on the maternal allele – possibly via dosage compensation [38]. Yet targeting of AID to the paternal allele did not influence the DNA methylation status on the maternal allele, since bisulfite analysis of Bi-4 showed no change in any of the mice analyzed (Figure 5E and Figure S5B). The paternal DMR DNA methylation status (Bi-2 and Bi-3) was also analyzed from embryos and placenta (Figure 6 and Figure S5C), and analogous to the results from the liver tissue, catalytic AID induced local DNA demethylation.

### AID-induced demethylation outside its target motif

We estimated the extent of AID-induced DNA demethylation of at least 1,000 bases at the H19 locus, as the paternal specific Bi-2 and Bi-3 are each about 500 bases long and were substantially demethylated. The upstream border of DNA demethylation could be situated near the 5′ part of the Bi-2 (further upstream are no polymorphisms to distinguish between maternal and paternal alleles); the downstream border is likely the G-repeats, inhibiting the DNA demethylation.

AID favors the hot spot motif WRC (A/T,A/G,C) and strongly disfavors SYC (cold spot - G/C, C/T, C) sequences for deamination [27], which is even more pronounced on a 5mC containing substrate [6]. Sequence context analysis of the Bi-2 demethylated CpGs showed that there was no difference in targeting cold spots or hot spots (Figure 5C; cold spot - green, hot spot - red), indicating that it is unlikely for AID to have targeted each and every CpG. During immune diversification, AID can be linked to the SP-BER pathway [16], while we [6] and others [11], [12], [39] have implicated SP-BER with AID-induced demethylation. Yet, this single 5mCpG targeting pathway has its limitations. The AID-induced demethylated Bi-2 & Bi-3 regions contained 88 5mCs among 582 dCs. In order for AID to target each CpG separately (followed by faithful DNA repair), 5279 independent deaminations would have had to occur (Figure S6), with each lesion being repaired by SP-BER. It is more likely that *in vivo* DNA deamination induced DNA demethylation proceeds via a processive DNA polymerase repair pathway (e.g. LP-BER or MMR), allowing for multiple CpG demethylations to occur from a single DNA lesion. Overall, these data indicated that the GAL4-AID-induced demethylation occurred early in mouse development and involves both processive (initiated from a single AID-induced lesion but leading to multiple demethylation events) and non-processive (one lesion one demethylation) DNA demethylation activity.

## Discussion

**Figure 7:**
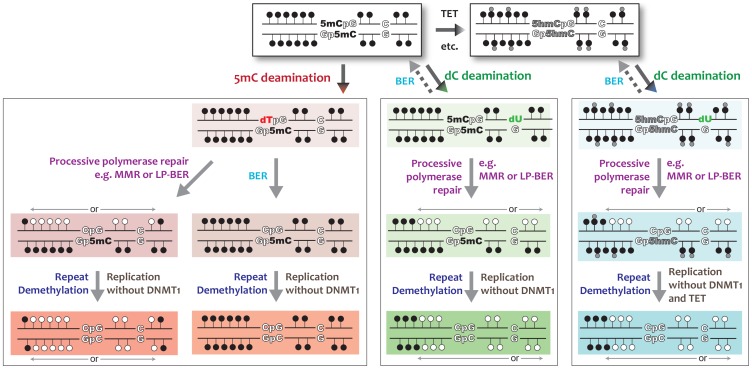
Model for lesion-induced DNA demethylation. Active DNA demethylation dependent on 5mC targeting. 5mC (filled circle) can be hydroxymethylated (5hmC - small grey circle) and further processed by the TET protein family (top right panel - grey). 5mC can be deaminated by the cytosine deaminase AID (bottom left panel - red). Deamination of 5mC results in thymine (dT - in red) and creates a dT:G mismatch that can be recognized by multiple DNA repair pathways, including BER or processive polymerase dependent repair. The processing of the dT:G mismatch by BER produces a single cytosine demethylation event, while processive polymerase dependent repair, in either direction, replaces long stretches of all bases, including 5mC, leading to multiple cytosine-demethylation events. Active DNA demethylation independent of 5mC targeting (central panel - green). AID targets and deaminates cytosine (white C:G), forming uracil (dU – in green) and leading to a dU:G mismatch. BER processing leads to the status quo (intact methylated DNA), while activation of a processive polymerase dependent repair, in either direction, replaces long stretches of all bases, including 5mC, leading to multiple cytosine-demethylation events. After AID and DNA repair induced demethylation on one strand, the complementary strand can be targeted as well and/or DNA replication can take place in the absence of DNMT1 activity. Active DNA demethylation of TET enzyme induced 5hmC can be independent of targeting (right panel - blue). AID targets and deaminates cytosine (white C:G), forming uracil (dU – in green) and leading to a dU:G mismatch. Processing as for untargeted 5mC demethylation with the outcome of replacing 5hmC with dC.

DNA instability plays a pivotal role in survival and evolution, with physical and chemical DNA damage being mutagenic, while at the same time controlled DNA alterations provide adaptation to environmental stress, either during meiosis or the development of the adaptive immune system. DNA methylation is the sum of DNA methylation and active/passive demethylation [2], [3], [40], with active removal of the methyl-mark requiring DNA base modification or removal of the modified-cytosine. The molecular processes activated to induce DNA demethylation will determine the extent and efficiency of the epigenetic change. Recent data have shown that single base demethylation can be achieved via different pathways [5], [6], [8], [11], while our current work proposes that a more efficient way for DNA demethylation can occur. By utilizing different DNA repair pathways, either UNG dependent or independent (coupled to processive DNA polymerizations), a single DNA lesion could induce multiple DNA demethylation events (Figure 7). At a targeted locus AID activity on cytosine (regardless of its methylation status) would induce DNA mismatches (dU:dG or dT:dG). dT:dG processing via BER would lead to a single DNA demethylation, while dU:dG processing via BER leads to the status quo. On the other hand, DNA repair utilizing a processive DNA polymerase (e.g. MMR or long-path BER) would lead to extensive removal of 5mC marks from a single lesion. MMR is known to replace up to 2 kb of ssDNA away from the lesion itself [41], [42], and if taking place near 5mCpGs then each 5mC would be replaced by cytosine within this stretch. This model would reduce the number of DNA damages required to induce DNA demethylation (Figure S6), increase efficiency and accuracy, and be in line with known AID-induced DNA repair pathways. Recent analysis of DNA methylation status immediately post-fertilization suggested processive DNA demethylation (possibly via LP-BER) to occur in the second phase of active DNA demethylation [43], mimicking our *in vitro* and *in vivo* findings and supporting our insights into the mechanisms of active DNA demethylation. Future work will determine if the findings form our model system and its DNA repair pathway choice will also be observed as pathways for AID induced demethylation *in vivo*.

The design of the IVR assay and *in vivo* targeting precludes analysis of how AID reaches its target as the GAL4 binding capacity vastly exceeds that of AID. In the B cell community it is well known that targeting of AID to the Ig locus is insufficient to explains AID's ability to induce a mutation rate that is 10^6^ fold above background mutations [19], [28], [44]. Even within the same cell an AID lesion can be repaired or lead to mutations depending on the chromatin context [45]. Hence the cellular milieu and chromatin context can be instructive for AID's effectiveness. Both of these aspects can be carefully controlled in our IVR assay and we are currently developing new tools and readouts to gain more insight. We are also looking at the mechanistic differences of how AID's activity in early developmental stages can lead to DNA demethylation [13], [43] or how AID can alter epithelial-mesenchymal transition (EMT) [46]. Interestingly, in B cells where AID is to induce DNA mutations AID seems to have less of an influence on the dynamics of DNA methylation [47].

### Insights from novel DNA damage resolution assays

Our systems allowed us to dissect the molecular mechanisms of DNA lesion resolution through genetics and biochemistry. Unlike previous *in vitro* systems for studying AID lesion resolution [48], [49], the IVR system utilizes the physiological DNA damaging activity of AID, thereby providing the first biochemical approach to study multiple aspects of AID-induced demethylation. Addition of Ugi and bio-dA to the IVR allowed DNA damage resolution to proceed either through the UNG-dependent BER pathway (Ugi sensitive) or through a processive DNA polymerase dependent repair pathway (MMR-like pathway - dA incorporation). This lesion channelling (BER vs MMR-like) is analogous to the *in vivo* DNA repair pathway choice of Ugi addition during Ig diversification [19]. The IVR systems results were recapitulated in the *in vivo* AID targeting work and provided additional evidence that DNA demethylation can proceed via various DNA repair pathways.

Aphidicolin is predominately used to inhibit replicative polymerases, but can also inhibit DNA pol delta, a polymerase associated with MMR. Therefore, the use of aphidicolin during the resolution phase (in the presence of Ugi) of the IVR suggests that the AID-induced dU:dG mismatch is not resolved via classical MMR. Interestingly, it has been suggested that AID-linked MMR activity during Ig diversification proceeds via a noncanonical MMR pathway [21], raising the possibility that AID-induced DNA demethylation can also involve non-classical DNA repair pathways. This possible ‘high-jacking’ of classical DNA repair factors for non-classical functions also occurs during AID-induced SHM [19]. A link of this interplay has been observed *in vivo*, as the CpG methylation status within the Ig locus can alter SHM outcome [50], [51]. Future work using the IVR and AID will also allow for the uncovering of the precise molecular mechanisms of how AID-induced demethylation can proceed during primordial germ cell formation [13], during pluripotency reprogramming [14], [15], and zebrafish development [12].

It is of course possible that other DNA damaging events (aside from AID-induced lesions) away from the 5mCpG, which can be repaired with processive DNA repair, such as DNA topoisomerase lesions, could serve as substrates for DNA demethylation. It is interesting to note that *in vivo* treatment of cells with topoisomerase inhibitors lead to DNA demethylation, rather than hypermethylation as observed for other DNA synthesis inhibitors [52].

### AID and TET dependent DNA demethylation

We and others have previously shown that AID is unable to deaminate 5hmC containing substrates [32], [53], and hence the proposed direct genetic links [54], [55] between AID and 5hmC were only speculative. Our discovery that AID-induced lesions at non-methylated cytosines can lead to active DNA demethylation provides an alternative indirect pathway for AID to resolve TET induced 5hmC modifications. As shown in Figure 7 (far right pathway), if the AID-induced uracil is present near a 5hmC, then upon processive polymerase dependent DNA repair 5hmC would be replaced with unmodified cytosine. If and to what extent this pathway has an *in vivo* physiological role is yet to be discovered.

### Conclusion

We have shown that local DNA demethylation, induced from a single DNA lesion (e.g. deamination of dC or 5mC), proceeded via at least two different efficient DNA repair pathways. We propose that this will also hold true for some aspects of global DNA demethylation, with pathway choice influencing the extent and efficiency of this mechanism. Depending on the lesion (e.g. base modification, mismatch, ssDNA nick), the genetic loci (intra-, intergenic), the chromatin state (e.g. DNA methylation, histone modification, polycomb complex association), or cellular milieu (e.g. B cell, germ cell) different DNA repair pathways will induce lesion resolution and DNA demethylation. Therefore, a single lesion may lead to single (via BER) or multiple (possibly via processive DNA polymerase dependent repair) 5mCpG demethylation. As epigenetics, including DNA (de)methylation, is becoming more relevant for understanding oncology, this work could have a direct impact on patient care. Clinical evidence already suggest a link between DNA methylation and DNA repair for drug efficacy, therefore the IVR and *in vivo* targeting could provide a means to identify new drug targets.

## Supporting Information

Figure S1 **Schematic of IVR on methylated substrate.** Schematic description of the in vitro assay using a methylated substrate, modified from [1]. Prior to the reaction the supercoiled plasmid is in vitro methylated by using the CpG DNA methyltransferase M.SssI. The methylated (filled lollipops) DNA plasmid containing GAL4 binding sites is incubated with a recombinant Gal4-AID fusion protein creating a dU lesion (green star). The supercoiling provides a region of dsDNA for GAL4 binding and a region of ssDNA for AID activity. Addition of frog egg extract (FE), containing topoisomerases, relaxes the substrate plasmid forming a dU:dG mismatch. The repair phase in the FE is carried out in the presence of biotinylated dCTP (bio-dC) or dATP (bio-dA) - (blue arrow), along with normal dNTPs. Repaired and biotinylated DNA is isolated via magnetic streptavidin beads. Prior to streptavidin isolation a small sample (input) is removed from the reaction. Eluted products and input are then subject to quantitative real-time PCR (red bar).
(PDF)

Figure S2 ***In vitro*** **methylation does not trigger IVR activity.** (A) The plasmid was in vitro methylated with the CpG methyltransferase M.SssI. The unmethylated plasmid (UM) was incubated with the buffer ingredients only (UM Buffer), mock methylated (Mock M) with the M.SssI only (- SAM) or the cofactor SAM only (- SssI), or methylated with all the components (M). The methylation status was monitored by digestion with the methyl sensitive enzyme BstUI and analysis on a 0.8% agarose gel, post-stained with SYBR safe. (B) Topology of an in vitro methylated plasmid. 0.5 µg of all of the different plasmids obtained in (A) were electrophoresed on 0.8% agarose gel for 10 h at 5 V/cm at 4°C. After migration the gel was soaked in 1x TBE containing 0.3 mg/ml ethidium bromide for 1 h and visualized with a Gel Doc (Bio-Rad). (C) Mock methylated plasmids are equivalent to unmethylated plasmids in the IVR. Unmethylated plasmid (UM), unmethylated plasmid containing methylation buffer (UM Buffer), and mock methylated plasmids (Mock M, -SAM or –SssI) were used in the IVR assay. The bars represent the ratio of the amount of recovered plasmids from reactions carried out in the presence of G-AID versus absence of G-AID (FE alone was set to 1). Error bars indicate ± standard deviation (SD, n = 3).
(PDF)

Figure S3 **FE activity on** ***dam*** **methylated adenine substrates.** IVR was performed with plasmids isolated from Dam+ bacteria. Briefly, the dam-methylated plasmid was incubated with the FE and after treatment the plasmid was either mock digested (/) or digested with MboI, DpnI and Sau3AI prior to isolation and qPCR amplification. The bars show ratio of cut versus uncut DNA (set to 1) after FE treatment.
(PDF)

Figure S4 **Assessment of mAID protein activity.** Wild type but not mutant mouse AID protein induces mutation in E. coli. The control empty vector (vector), mouse AID wild type (mAID), mouse AID mutant D89 - C147R (mutations in TG 7 line), and mouse AID mutant E58G (mutations in TG 8 line) were transformed into bacteria (BW310 - ungΔ - [2]). Protein expression was induced with 0.4 mM IPTG for 14 h at 37°C. The bacteria were plated on low salt LB agar plates to assess viability or on rifampicin plates to determine the mutation frequency in the rpoB gene. For each sample the number of Rif^R^ clones per 10^9^ viable cells is plotted [25].
(PDF)

Figure S5 **Table summary of G-AID transgenic demethylation.** (A) Table showing the summary of the DNA methylation status at the Bi-2 and Bi-3 regions after bisulfite treatment of DNA from fetal liver. Data summarized from Figure 5. The number in bracket is the number of transgenic mice analyzed. (B) Table showing the summary of the DNA methylation status at the Bi-4 region after bisulfite treatment of DNA from fetal liver. Data summarized from Figure 5. (C) Table showing the summary of the DNA methylation status at the Bi-2 and Bi-3 regions after bisulfite treatment of DNA from Embryos and Placentas (E12.5). Data summarized from Figure 6. The number in bracket is the number of transgenic mice analyzed.
(PDF)

Figure S6 **Theoretical approximation of the number of deaminations required for demethylation based on single BER events.** The transgenic mouse H19 locus is schematically drawn with the GAL4 binding sites (UAS) in the centre, surrounded by the bisulfite sequenced flanking (filled circle - 5mC) regions. GAL4-AID (circle-triangle) is bound at the UAS with arrows representing individual DNA deaminations, leading to complete demethylation (open circle). Number of total dCs (582) and 5mC (88) in this region is indicated, followed by a set of assumptions for the calculation. After n deaminations, the probability for a single target of being never hit is (581/582)∧n and its probability of being hit at least once is 1-(581/582)∧n. Deriving at a formula representing the number of deaminations that have to occur in order to have ‘hit’ 88 5mC in 582 dC with 99% confidence.
(PDF)

Table S1 Primers used for bisulfite analysis. List of primers used in the bisulfite analysis.
(DOC)

References S1 (DOC)

## References

[1] Bird A (2002) DNA methylation patterns and epigenetic memory. Genes Dev 16: 6–21. 11782440 10.1101/gad.947102

[2] Wu SC, Zhang Y (2010) Active DNA demethylation: many roads lead to Rome. Nat Rev Mol Cell Biol 11: 607–620. 20683471 10.1038/nrm2950 PMC3711520

[3] Franchini D-M, Schmitz K-M, Petersen-Mahrt SK (2012) 5-Methylcytosine DNA Demethylation: More Than Losing a Methyl Group. Annu Rev Genet. 10.1146/annurev-genet-110711-155451 22974304

[4] Gehring M, Reik W, Henikoff S (2009) DNA demethylation by DNA repair. Trends Genet 25: 82–90. 19144439 10.1016/j.tig.2008.12.001

[5] Hajkova P, Jeffries SJ, Lee C, Miller N, Jackson SP, et al (2010) Genome-wide reprogramming in the mouse germ line entails the base excision repair pathway. Science 329: 78–82. 20595612 10.1126/science.1187945 PMC3863715

[6] Morgan HD, Dean W, Coker HA, Reik W, Petersen-Mahrt SK (2004) Activation-induced cytidine deaminase deaminates 5-methylcytosine in DNA and is expressed in pluripotent tissues: implications for epigenetic reprogramming. J Biol Chem 279: 52353–52360. 15448152 10.1074/jbc.M407695200

[7] Ko M, Huang Y, Jankowska AM, Pape UJ, Tahiliani M, et al (2010) Impaired hydroxylation of 5-methylcytosine in myeloid cancers with mutant TET2. Nature 468: 839–843. 21057493 10.1038/nature09586 PMC3003755

[8] Tahiliani M, Koh KP, Shen Y, Pastor WA, Bandukwala H, et al (2009) Conversion of 5-methylcytosine to 5-hydroxymethylcytosine in mammalian DNA by MLL partner TET1. Science 324: 930–935. 19372391 10.1126/science.1170116 PMC2715015

[9] Ito S, D'Alessio AC, Taranova OV, Hong K, Sowers LC, et al (2010) Role of Tet proteins in 5mC to 5hmC conversion, ES-cell self-renewal and inner cell mass specification. Nature 466: 1129–1133. 20639862 10.1038/nature09303 PMC3491567

[10] He Y-F, Li B-Z, Li Z, Liu P, Wang Y, et al.. (2011) Tet-Mediated Formation of 5-Carboxylcytosine and Its Excision by TDG in Mammalian DNA. Science. 10.1126/science.1210944 PMC3462231 21817016

[11] Cortázar D, Kunz C, Selfridge J, Lettieri T, Saito Y, et al (2011) Embryonic lethal phenotype reveals a function of TDG in maintaining epigenetic stability. Nature 470: 419–423. 21278727 10.1038/nature09672

[12] Rai K, Huggins IJ, James SR, Karpf AR, Jones DA, et al (2008) DNA demethylation in zebrafish involves the coupling of a deaminase, a glycosylase, and gadd45. Cell 135: 1201–1212. 19109892 10.1016/j.cell.2008.11.042 PMC2629358

[13] Popp C, Dean W, Feng S, Cokus SJ, Andrews S, et al (2010) Genome-wide erasure of DNA methylation in mouse primordial germ cells is affected by AID deficiency. Nature 463: 1101–1105. 20098412 10.1038/nature08829 PMC2965733

[14] Bhutani N, Brady JJ, Damian M, Sacco A, Corbel SY, et al (2010) Reprogramming towards pluripotency requires AID-dependent DNA demethylation. Nature 463: 1042–1047. 20027182 10.1038/nature08752 PMC2906123

[15] Kumar R, DiMenna L, Schrode N, Liu TC, Franck P, et al (2013) AID stabilizes stem-cell phenotype by removing epigenetic memory of pluripotency genes. Nature 500: 89–92. 23803762 10.1038/nature12299 PMC3762466

[16] Petersen-Mahrt S (2005) DNA deamination in immunity. Immunol Rev 203: 80–97. 15661023 10.1111/j.0105-2896.2005.00232.x

[17] Muramatsu M, Kinoshita K, Fagarasan S, Yamada S, Shinkai Y, et al (2000) Class switch recombination and hypermutation require activation-induced cytidine deaminase (AID), a potential RNA editing enzyme. Cell 102: 553–563. 11007474 10.1016/s0092-8674(00)00078-7

[18] Revy P, Muto T, Levy Y, Geissmann F, Plebani A, et al (2000) Activation-induced cytidine deaminase (AID) deficiency causes the autosomal recessive form of the Hyper-IgM syndrome (HIGM2). Cell 102: 565–575. 11007475 10.1016/s0092-8674(00)00079-9

[19] Di Noia JM, Neuberger MS (2007) Molecular mechanisms of antibody somatic hypermutation. Annu Rev Biochem 76: 1–22. 17328676 10.1146/annurev.biochem.76.061705.090740

[20] Liu M, Schatz DG (2009) Balancing AID and DNA repair during somatic hypermutation. Trends Immunol 30: 173–181. 19303358 10.1016/j.it.2009.01.007

[21] Peña-Diaz J, Bregenhorn S, Ghodgaonkar M, Follonier C, Artola-Borán M, et al (2012) Noncanonical mismatch repair as a source of genomic instability in human cells. Mol Cell 47: 669–680. 22864113 10.1016/j.molcel.2012.07.006

[22] Franchini DM, Incorvaia E, Rangam G, Coker HA, Petersen-Mahrt SK (2013) Simultaneous In Vitro Characterisation of DNA Deaminase Function and Associated DNA Repair Pathways. PLoS One 8: e82097. 24349193 10.1371/journal.pone.0082097 PMC3857227

[23] Brenner C, Deplus R, Didelot C, Loriot A, Vire E, et al (2005) Myc represses transcription through recruitment of DNA methyltransferase corepressor. EMBO J 24: 336–346. 15616584 10.1038/sj.emboj.7600509 PMC545804

[24] Olek A, Oswald J, Walter J (1996) A modified and improved method for bisulphite based cytosine methylation analysis. Nucleic Acids Res 24: 5064–5066. 9016686 10.1093/nar/24.24.5064 PMC146326

[25] Coker HA, Morgan HD, Petersen-Mahrt SK (2006) Genetic and in vitro assays of DNA deamination. Methods in Enzymology 408: 156–170. 16793368 10.1016/S0076-6879(06)08010-4

[26] Trenz K, Errico A, Costanzo V (2008) Plx1 is required for chromosomal DNA replication under stressful conditions. EMBO J 27: 876–885. 18309293 10.1038/emboj.2008.29 PMC2265110

[27] Bransteitter R, Pham P, Scharff MD, Goodman MF (2003) Activation-induced cytidine deaminase deaminates deoxycytidine on single-stranded DNA but requires the action of RNase. Proc Natl Acad Sci U S A 100: 4102–4107. 12651944 10.1073/pnas.0730835100 PMC153055

[28] Willmann KL, Milosevic S, Pauklin S, Schmitz KM, Rangam G, et al (2012) A role for the RNA pol II-associated PAF complex in AID-induced immune diversification. The Journal of experimental medicine 209: 2099–2111. 23008333 10.1084/jem.20112145 PMC3478926

[29] Dickerson SK, Market E, Besmer E, Papavasiliou FN (2003) AID mediates hypermutation by deaminating single stranded DNA. J Exp Med 197: 1291–1296. 12756266 10.1084/jem.20030481 PMC2193777

[30] Barnes DE, Lindahl T (2004) Repair and genetic consequences of endogenous DNA base damage in mammalian cells. Annu Rev Genet 38: 445–476. 15568983 10.1146/annurev.genet.38.072902.092448

[31] Karran P, Cone R, Friedberg EC (1981) Specificity of the bacteriophage PBS2 induced inhibitor of uracil-DNA glycosylase. Biochemistry 20: 6092–6096. 6796110 10.1021/bi00524a027

[32] Rangam G, Schmitz KM, Cobb AJ, Petersen-Mahrt SK (2012) AID enzymatic activity is inversely proportional to the size of cytosine C5 orbital cloud. PLoS One 7: e43279. 22916236 10.1371/journal.pone.0043279 PMC3423351

[33] Murrell A, Heeson S, Reik W (2004) Interaction between differentially methylated regions partitions the imprinted genes Igf2 and H19 into parent-specific chromatin loops. Nat Genet 36: 889–893. 15273689 10.1038/ng1402

[34] Geisberger R, Rada C, Neuberger MS (2009) The stability of AID and its function in class-switching are critically sensitive to the identity of its nuclear-export sequence. Proc Natl Acad Sci U S A 106: 6736–6741. 19351893 10.1073/pnas.0810808106 PMC2672500

[35] Patenaude AM, Orthwein A, Hu Y, Campo VA, Kavli B, et al (2009) Active nuclear import and cytoplasmic retention of activation-induced deaminase. Nat Struct Mol Biol 16: 517–527. 19412186 10.1038/nsmb.1598

[36] Barreto V, Reina-San-Martin B, Ramiro AR, McBride KM, Nussenzweig MC (2003) C-terminal deletion of AID uncouples class switch recombination from somatic hypermutation and gene conversion. Mol Cell 12: 501–508. 14536088 10.1016/s1097-2765(03)00309-5

[37] Ta VT, Nagaoka H, Catalan N, Durandy A, Fischer A, et al (2003) AID mutant analyses indicate requirement for class-switch-specific cofactors. Nat Immunol 4: 843–848. 12910268 10.1038/ni964

[38] Chow JC, Heard E (2010) Nuclear organization and dosage compensation. Cold Spring Harbor Perspectives in Biology 2: a000604. 20943757 10.1101/cshperspect.a000604 PMC2964184

[39] Zhu J-K (2009) Active DNA demethylation mediated by DNA glycosylases. Annu Rev Genet 43: 143–166. 19659441 10.1146/annurev-genet-102108-134205 PMC3137514

[40] Bhutani N, Burns DM, Blau HM (2011) DNA demethylation dynamics. Cell 146: 866–872. 21925312 10.1016/j.cell.2011.08.042 PMC3236603

[41] Constantin N, Dzantiev L, Kadyrov FA, Modrich P (2005) Human mismatch repair: reconstitution of a nick-directed bidirectional reaction. J Biol Chem 280: 39752–39761. 16188885 10.1074/jbc.M509701200 PMC1435381

[42] Jiricny J (2006) The multifaceted mismatch-repair system. Nat Rev Mol Cell Biol 7: 335–346. 16612326 10.1038/nrm1907

[43] Santos F, Peat J, Burgess H, Rada C, Reik W, et al (2013) Active demethylation in mouse zygotes involves cytosine deamination and base excision repair. Epigenetics & chromatin 6: 39. 24279473 10.1186/1756-8935-6-39 PMC4037648

[44] Schmitz K-M, Petersen-Mahrt SK (2012) AIDing the immune system-DIAbolic in cancer. Semin Immunol 24: 241–245. 22841422 10.1016/j.smim.2012.07.001

[45] Liu M, Duke JL, Richter DJ, Vinuesa CG, Goodnow CC, et al (2008) Two levels of protection for the B cell genome during somatic hypermutation. Nature 451: 841–845. 18273020 10.1038/nature06547

[46] Munoz DP, Lee EL, Takayama S, Coppe JP, Heo SJ, et al (2013) Activation-induced cytidine deaminase (AID) is necessary for the epithelial-mesenchymal transition in mammary epithelial cells. Proc Natl Acad Sci U S A 110: E2977–2986. 23882083 10.1073/pnas.1301021110 PMC3740878

[47] Fritz EL, Rosenberg BR, Lay K, Mihailovic A, Tuschl T, et al (2013) A comprehensive analysis of the effects of the deaminase AID on the transcriptome and methylome of activated B cells. Nature immunology 14: 749–755. 23708250 10.1038/ni.2616 PMC3688651

[48] Pham P, Zhang K, Goodman MF (2008) Hypermutation at A/T sites during G.U mismatch repair in vitro by human B-cell lysates. J Biol Chem 283: 31754–31762. 18786917 10.1074/jbc.M805524200 PMC2581569

[49] Schanz S, Castor D, Fischer F, Jiricny J (2009) Interference of mismatch and base excision repair during the processing of adjacent U/G mispairs may play a key role in somatic hypermutation. Proc Natl Acad Sci U S A 106: 5593–5598. 19307563 10.1073/pnas.0901726106 PMC2659717

[50] Fraenkel S, Mostoslavsky R, Novobrantseva TI, Pelanda R, Chaudhuri J, et al (2007) Allelic ‘choice’ governs somatic hypermutation in vivo at the immunoglobulin kappa-chain locus. Nat Immunol 8: 715–722. 17546032 10.1038/ni1476

[51] Jolly CJ, Neuberger MS (2001) Somatic hypermutation of immunoglobulin kappa transgenes: association of mutability with demethylation. Immunol Cell Biol 79: 18–22. 11168618 10.1046/j.1440-1711.2001.00968.x

[52] Nyce J, Liu L, Jones PA (1986) Variable effects of DNA-synthesis inhibitors upon DNA methylation in mammalian cells. Nucleic Acids Research 14: 4353–4367. 3086840 10.1093/nar/14.10.4353 PMC339866

[53] Nabel CS, Jia H, Ye Y, Shen L, Goldschmidt HL, et al (2012) AID/APOBEC deaminases disfavor modified cytosines implicated in DNA demethylation. Nature chemical biology 8: 751–758. 22772155 10.1038/nchembio.1042 PMC3427411

[54] Cortellino S, Xu J, Sannai M, Moore R, Caretti E, et al (2011) Thymine DNA glycosylase is essential for active DNA demethylation by linked deamination-base excision repair. Cell 146: 67–79. 21722948 10.1016/j.cell.2011.06.020 PMC3230223

[55] Guo JU, Su Y, Zhong C, Ming GL, Song H (2011) Hydroxylation of 5-methylcytosine by TET1 promotes active DNA demethylation in the adult brain. Cell 145: 423–434. 21496894 10.1016/j.cell.2011.03.022 PMC3088758

