# Supplementary figures and images for: Processive DNA Demethylation via DNA Deaminase-Induced Lesion Resolution
Source: PLoS One. 2014 Jul 15;9(7):e97754. doi: 10.1371/journal.pone.0097754 (PMC4098905; doi:10.1371/journal.pone.0097754)

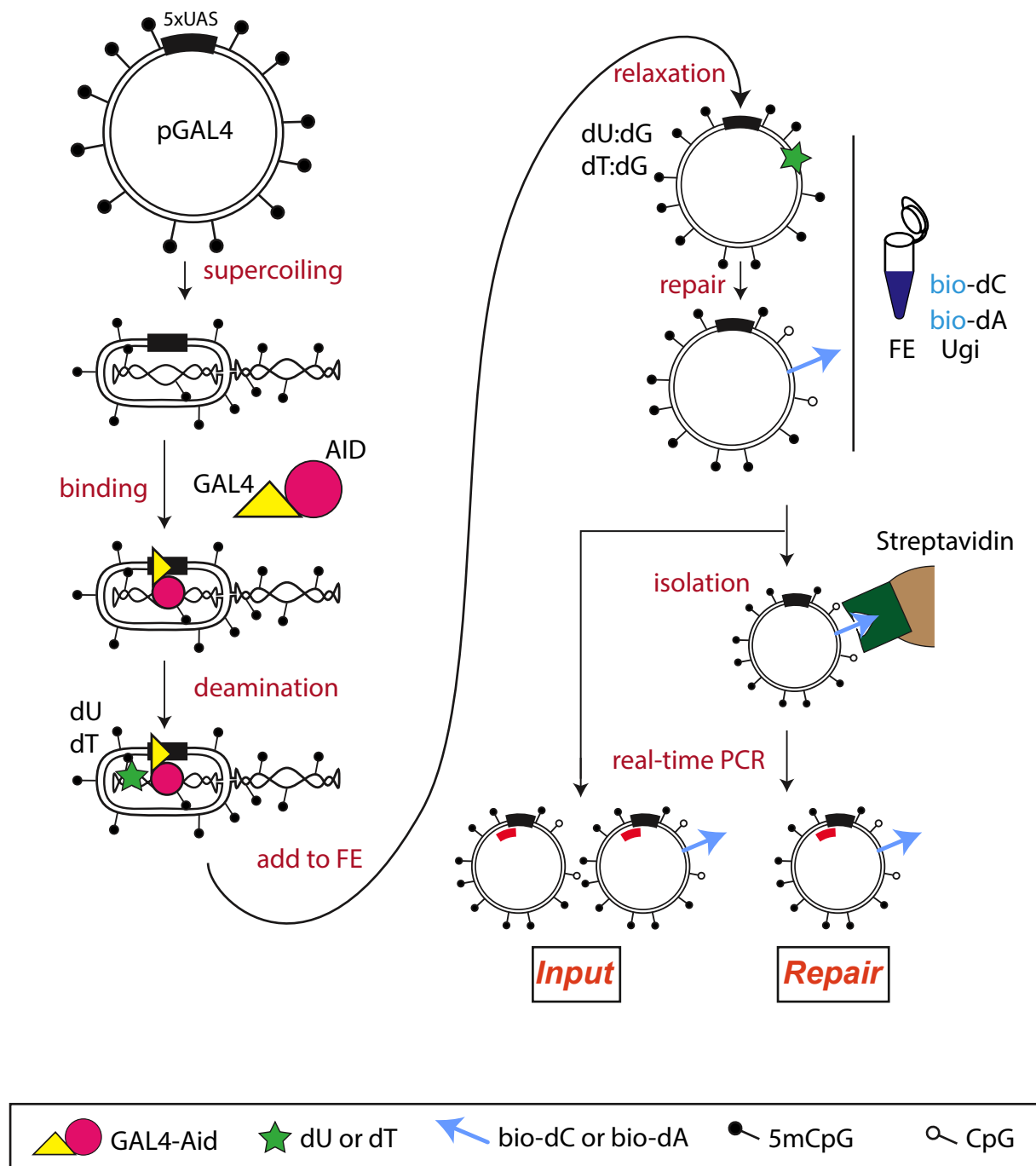

Supplement: Figure S1 — Schematic of IVR on methylated substrate. Schematic description of the in vitro assay using a methylated substrate, modified from [1]. Prior to the reaction the supercoiled plasmid is in vitro methylated by using the CpG DNA methyltransferase M.SssI. The methylated (filled lollipops) DNA plasmid containing GAL4 binding sites is incubated with a recombinant Gal4-AID fusion protein creating a dU lesion (green star). The supercoiling provides a region of dsDNA for GAL4 binding and a region of ssDNA for AID activity. Addition of frog egg extract (FE), containing topoisomerases, relaxes the substrate plasmid forming a dU:dG mismatch. The repair phase in the FE is carried out in the presence of biotinylated dCTP (bio-dC) or dATP (bio-dA) - (blue arrow), along with normal dNTPs. Repaired and biotinylated DNA is isolated via magnetic streptavidin beads. Prior to streptavidin isolation a small sample (input) is removed from the reaction. Eluted products and input are then subject to quantitative real-time PCR (red bar). (PDF) [file pone.0097754.s001.pdf]

**A**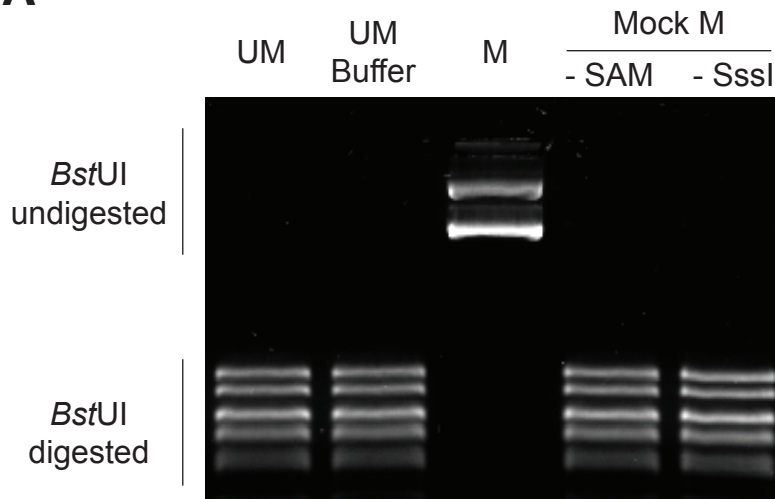**B**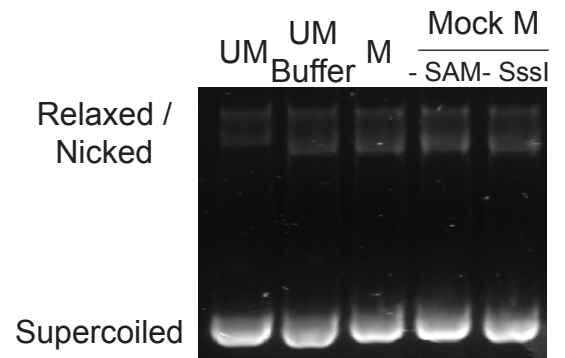**C**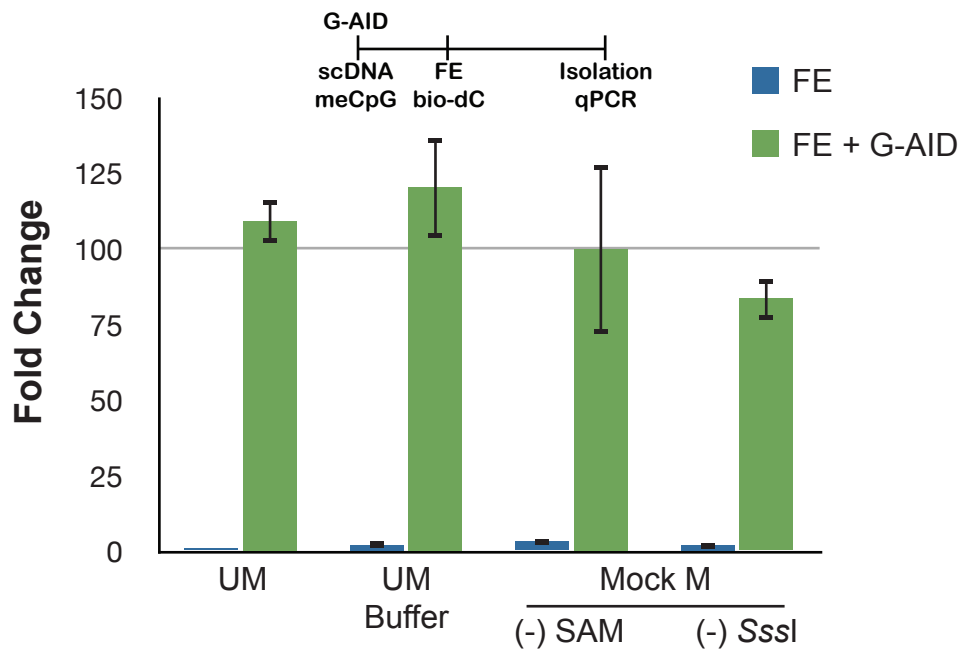

Supplement: Figure S2 — In vitro methylation does not trigger IVR activity. (A) The plasmid was in vitro methylated with the CpG methyltransferase M.SssI. The unmethylated plasmid (UM) was incubated with the buffer ingredients only (UM Buffer), mock methylated (Mock M) with the M.SssI only (- SAM) or the cofactor SAM only (- SssI), or methylated with all the components (M). The methylation status was monitored by digestion with the methyl sensitive enzyme BstUI and analysis on a 0.8% agarose gel, post-stained with SYBR safe. (B) Topology of an in vitro methylated plasmid. 0.5 µg of all of the different plasmids obtained in (A) were electrophoresed on 0.8% agarose gel for 10 h at 5 V/cm at 4°C. After migration the gel was soaked in 1x TBE containing 0.3 mg/ml ethidium bromide for 1 h and visualized with a Gel Doc (Bio-Rad). (C) Mock methylated plasmids are equivalent to unmethylated plasmids in the IVR. Unmethylated plasmid (UM), unmethylated plasmid containing methylation buffer (UM Buffer), and mock methylated plasmids (Mock M, -SAM or –SssI) were used in the IVR assay. The bars represent the ratio of the amount of recovered plasmids from reactions carried out in the presence of G-AID versus absence of G-AID (FE alone was set to 1). Error bars indicate ± standard deviation (SD, n = 3). (PDF) [file pone.0097754.s002.pdf]

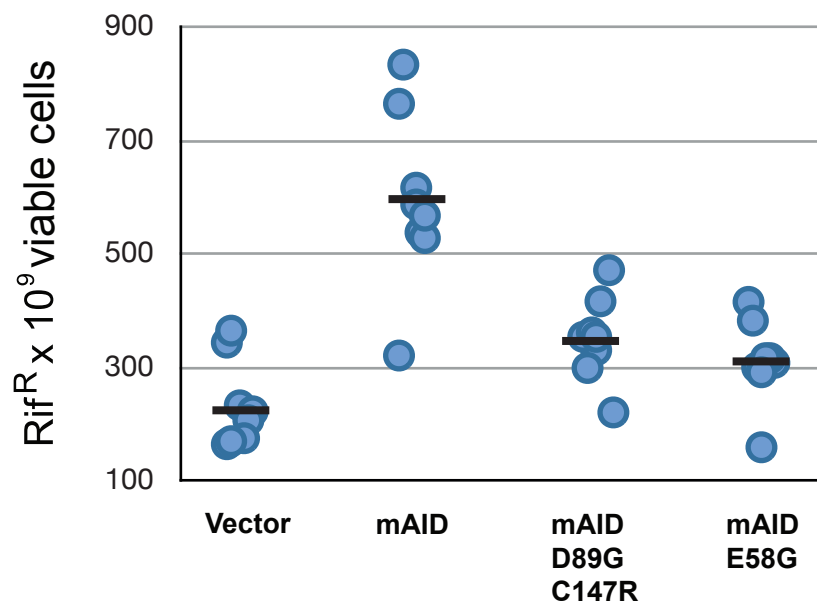

Supplement: Figure S4 — Assessment of mAID protein activity. Wild type but not mutant mouse AID protein induces mutation in E. coli. The control empty vector (vector), mouse AID wild type (mAID), mouse AID mutant D89 - C147R (mutations in TG 7 line), and mouse AID mutant E58G (mutations in TG 8 line) were transformed into bacteria (BW310 - ungΔ - [2]). Protein expression was induced with 0.4 mM IPTG for 14 h at 37°C. The bacteria were plated on low salt LB agar plates to assess viability or on rifampicin plates to determine the mutation frequency in the rpoB gene. For each sample the number of RifR clones per 109 viable cells is plotted [25]. (PDF) [file pone.0097754.s004.pdf]
