# Supplementary material for: Processive DNA Demethylation via DNA Deaminase-Induced Lesion Resolution
Source: PLoS One. 2014 Jul 15;9(7):e97754. doi: 10.1371/journal.pone.0097754 (PMC4098905; doi:10.1371/journal.pone.0097754)
Supplement: Figure S3 — FE activity on dam methylated adenine substrates. IVR was performed with plasmids isolated from Dam+ bacteria. Briefly, the dam-methylated plasmid was incubated with the FE and after treatment the plasmid was either mock digested (/) or digested with MboI, DpnI and Sau3AI prior to isolation and qPCR amplification. The bars show ratio of cut versus uncut DNA (set to 1) after FE treatment. (PDF) [file pone.0097754.s003.pdf]

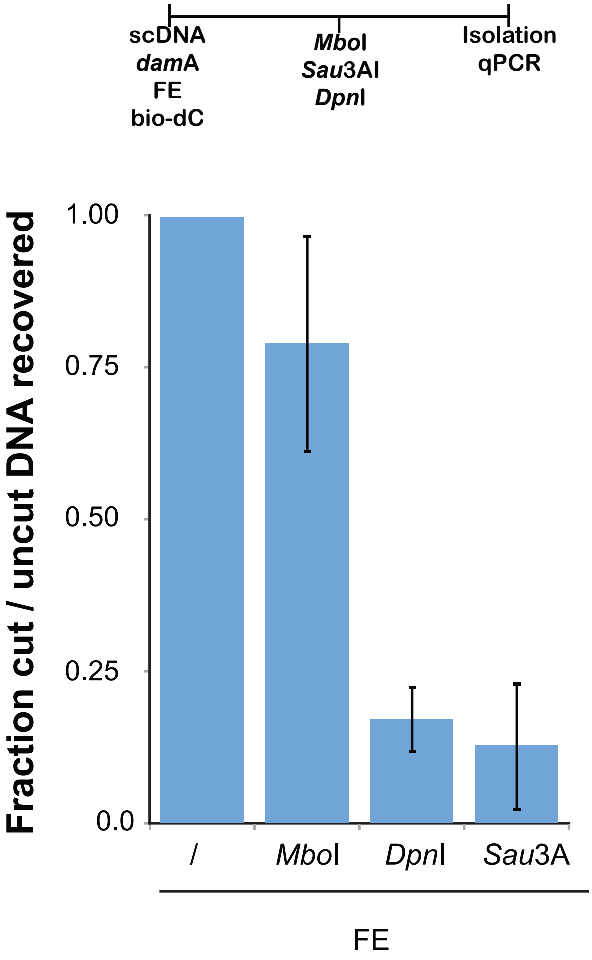

For calculating values in Figure 3B

$$\textbf{\% Recovery to uncut} = \frac{[\text{AID \& FE + MboI}]}{[\text{AID \& FE (/)}]} \times \frac{[\text{FE (/)}]}{[\text{FE + MboI}]}$$

For calculating values in Figure 3C

$$\textbf{\% Recovery to uncut} = \frac{[\text{AID \& FE + DpnI}]}{[\text{AID \& FE (/)}]} \times \frac{[\text{FE + Sau3A}]}{[\text{FE + DpnI}]}$$

Franchini et al., Supplementary Figure S3
