# Supplementary material for: Processive DNA Demethylation via DNA Deaminase-Induced Lesion Resolution
Source: PLoS One. 2014 Jul 15;9(7):e97754. doi: 10.1371/journal.pone.0097754 (PMC4098905; doi:10.1371/journal.pone.0097754)
Supplement: Figure S5 — Table summary of G-AID transgenic demethylation. (A) Table showing the summary of the DNA methylation status at the Bi-2 and Bi-3 regions after bisulfite treatment of DNA from fetal liver. Data summarized from Figure 5. The number in bracket is the number of transgenic mice analyzed. (B) Table showing the summary of the DNA methylation status at the Bi-4 region after bisulfite treatment of DNA from fetal liver. Data summarized from Figure 5. (C) Table showing the summary of the DNA methylation status at the Bi-2 and Bi-3 regions after bisulfite treatment of DNA from Embryos and Placentas (E12.5). Data summarized from Figure 6. The number in bracket is the number of transgenic mice analyzed. (PDF) [file pone.0097754.s005.pdf]

A

Neonatal Liver	mouse	Bi-2	Bi-3
Gal4-AID	TG 4 (2)	UnMet	UnMet
Gal4-AID	TG 5 (4)	UnMet	UnMet
Gal4-ΔAID1	TG 7 (3)	Met	N.D.
Gal4-Myc	TG 1 (3)	Met	Met

B

Neonatal Liver	mouse	Bi-4
Gal4-AID	TG 4	UnMet
Gal4-AID	TG 5	UnMet
Gal4-ΔAID1	TG 7	UnMet
Gal4-Myc	TG 1	UnMet

C

Embryo & Placenta (E12.5)	mouse	Bi-2	Bi-3
Gal4-AID	TG 5 (3)	UnMet	N.D.
Gal4-ΔAID1	TG 8 (3)	Met	Met

Franchini et al., Supplematal Figure S5
