# Supplementary material for: Processive DNA Demethylation via DNA Deaminase-Induced Lesion Resolution
Source: PLoS One. 2014 Jul 15;9(7):e97754. doi: 10.1371/journal.pone.0097754 (PMC4098905; doi:10.1371/journal.pone.0097754)
Supplement: Figure S6 — Theoretical approximation of the number of deaminations required for demethylation based on single BER events. The transgenic mouse H19 locus is schematically drawn with the GAL4 binding sites (UAS) in the centre, surrounded by the bisulfite sequenced flanking (filled circle - 5mC) regions. GAL4-AID (circle-triangle) is bound at the UAS with arrows representing individual DNA deaminations, leading to complete demethylation (open circle). Number of total dCs (582) and 5mC (88) in this region is indicated, followed by a set of assumptions for the calculation. After n deaminations, the probability for a single target of being never hit is (581/582)∧n and its probability of being hit at least once is 1-(581/582)∧n. Deriving at a formula representing the number of deaminations that have to occur in order to have ‘hit’ 88 5mC in 582 dC with 99% confidence. (PDF) [file pone.0097754.s006.pdf]

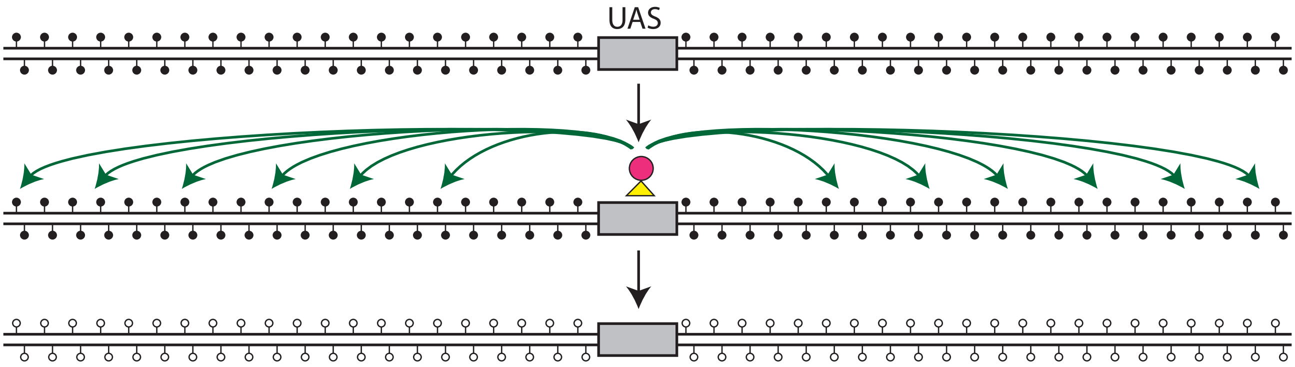

582 Cs (5mC + dC)
88 5mC

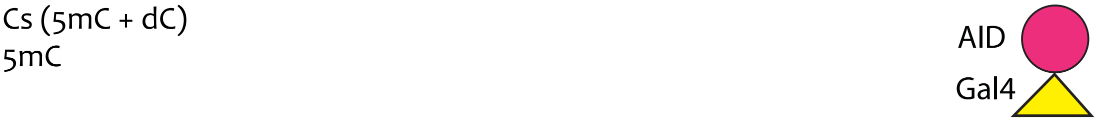

Assumption:

1. There is no sequence preference of targeting any cytosine.
2. There is no preference for dC vs 5mC.
3. There is no 'memory' of which meC has been changed to a dC.
   (repeatedly hitting the same dC [or 5mC converted] is possible)
4. We want to achieve 99 % demethylation (i.e. probability of 99%).
5. The chance distribution across the locus is a simple binomial.

$$1-\left(\frac{581}{582}\right)^{x} = \sqrt[88]{0.99} \longrightarrow 1-\sqrt[88]{0.99} = \left(\frac{581}{582}\right)^{x} \longrightarrow \frac{\ln\left(1-\sqrt[88]{0.99}\right)}{\ln\left(\frac{581}{582}\right)} = x$$

$$5279 = x$$

As indicated before, assumption 1) is not fully correct, since AID prefers WRC as a sequence context. Assumption 2) is also not fully correct, as *in vitro* AID deaminates dC 3 - 10 times more efficiently then 5mC [3].

These calculations do not include diffusion rates, BER repair efficiency, chromatin modifications, proof reading etc.

Franchini, et al. Supplementary Figure S6
