# Supplementary material for: Processive DNA Demethylation via DNA Deaminase-Induced Lesion Resolution
Source: PLoS One. 2014 Jul 15;9(7):e97754. doi: 10.1371/journal.pone.0097754 (PMC4098905; doi:10.1371/journal.pone.0097754)
Supplement: Table S1 — Primers used for bisulfite analysis. List of primers used in the bisulfite analysis. (DOC) [file pone.0097754.s007.doc]

Supplemental Table S1

| Bi2 |  |
| --- | --- |
| outer |  |
| sense | 5’-TGTGGTTTATTATAGGAAGGTATAGA-3’ |
| antisense | 5’-CAACCAAACTAACTTAACTACAAATC-3’ |
| inner |  |
| sense | 5’-GTAAGATGTGTGTATTTTTGGAAT-3’ |
| antisense | 5’-CAACCAAACTAACTTAACTACAAATC-3’ |
|  |  |
| Bi3 |  |
| outer |  |
| sense | 5’-TTTGTAGTTAAGTTAGTTTGGTTGG-3’ |
| antisense | 5’-CACTATACCCTCCCTACTATACCC-3’ |
| inner |  |
| sense | 5’-AAGTTATATTAAGGGTTATTGAATATG-3’ |
| antisense | 5’-TATATCCCCCACTATACCCACACTA-3’ |
|  |  |
| Bi4 |  |
| outer |  |
| sense | 5’-GTAAGATGTGTGTATTTTTGGAATG-3’ |
| antisense | 5’-AATCCCTAACTTCTCCTAATCTCTA-3’ |
| inner |  |
| sense | 5’-TATTTAATGTTTATAAGGGTTATGG-3' |
| antisense | 5’-ACTTTACAACTTCTTACTACAATTCC-3’ |
