# Supplementary material for: Processive DNA Demethylation via DNA Deaminase-Induced Lesion Resolution
Source: PLoS One. 2014 Jul 15;9(7):e97754. doi: 10.1371/journal.pone.0097754 (PMC4098905; doi:10.1371/journal.pone.0097754)
Supplement: References S1 — (DOC) [file pone.0097754.s008.doc]

Supporting Info Bibliography

1. Franchini DM, Incorvaia E, Rangam G, Coker HA, Petersen-Mahrt SK (2013) Simultaneous In Vitro Characterisation of DNA Deaminase Function and Associated DNA Repair Pathways. PLoS One 8: e82097.

2. Coker HA, Morgan HD, Petersen-Mahrt SK (2006) Genetic and in vitro assays of DNA deamination. Methods in Enzymology 408: 156-170.
